# Estimation of continuous elbow joint movement based on human physiological structure

**DOI:** 10.1186/s12938-019-0653-2

**Published:** 2019-03-20

**Authors:** Kexiang Li, Jianhua Zhang, Xuan Liu, Minglu Zhang

**Affiliations:** 1 School of Mechanical Engineering, Hebei University of Technology, Tianjin, 300130 China; 2 State Key Laboratory of Robotics, Shenyang Institute of Automation, Chinese Academy of Sciences, Shenyang, 110016 China

**Keywords:** Intention recognition, Elbow movement, Upper-limb physiological structure, Biomechanical, Surface electromyography, Genetic algorithm

## Abstract

**Objective:**

Human intention recognition technology plays a vital role in the application of robotic exoskeletons and powered exoskeletons. However, the precise estimation of the continuous motion of each joint represents a major challenge. In the current study, we present a method for estimating continuous elbow joint movement.

**Methods:**

We developed a novel approach for estimating the elbow joint angle based on human physiological structure. We used surface electromyography signals to analyze the biomechanical properties of the muscle and combined it with physiological structure to achieve a model for estimating continuous motion. And a genetic algorithm was used to optimize unknown parameters.

**Results:**

We performed extensive trials to verify the generalizability and effectiveness of this method. The trial types included elbow joint motion with single cycle trials, typical cycle trials, gradually increasing amplitude trials, and random movement trials for handheld loads of 1.25 and 2.5 kg. The results revealed that the average root-mean-square errors ranged from 0.12 to 0.26 rad, reflecting an appropriate level of estimation accuracy.

**Conclusion:**

Establishing a reasonable physiological model and applying an efficient optimization algorithm enabled more accurate estimation of the joint angle. The proposed method provides a theoretical foundation for robotic exoskeletons and powered exoskeletons to understand the intentions of human continuous motion.

## Background

In recent years, robotic exoskeletons have been widely applied in assisting the human body. However, human intention recognition technology remains a bottleneck that restricts the development of robotic exoskeletons. The main methods of intention recognition for human motion are based on human bioelectrical signals and mechanical sensor signals [1]. Human bioelectrical signals are potentials excited by neurons that carry human behavioral information when transmitted to related tissues/organs, directly reflecting human intentions. These signals can be collected from the human body via specialized electrodes, and motion intention can be judged by analyzing the signals. These methods include physiological models, classification and regression techniques, which have been used to estimate motion intention [2]. Currently available mechanical sensors mainly include angle sensors, inertial units and force sensors, which can be used to collect motion information such as angle, velocity, acceleration and ground reaction force during limb swing to judge the current motion state. However, mechanical sensor signals have a large time delay compared with bioelectrical signals, which can affect real-time recognition results. Using surface electromyography (sEMG) as incoming signals to realize a ‘friendly’ human–robot interface (HRI) for robotic exoskeletons is a simple method [3–5]. Previous studies of human intention identified by sEMG have largely focused on the classification of human actions for HRI systems. However, this method has only been used to predict a small number of discrete movements of the human body. Using the predicted results of this method to control a robot cannot achieve continuous movement that is comparable to that of human joints. Moreover, it cannot guarantee that the movement of the robot and human are closely matched [6, 7]. However, employing sEMG to directly measure a person’s intent during continuous motion has become an important area of research [6, 8]. For the type of continuous movement examined in the present study, human intent must be recognized from sEMG in a continuous manner, instead of discretely. In addition, the flexibility of human joints plays an important role in daily life, particularly in cases of high-precision poses with rapid switching, which reflect the superiority of joint flexibility. The current study presents a method for estimating continuous motion of the elbow joint based on the physiological structure of the upper limb, which is a method utilizing sEMG to determine human intent.

Previous studies have adopted many methods to estimate the continuous motion of human joints on the basis of sEMG. These studies have generally used muscle physiological mechanics to establish a joint dynamics model with sEMG data as the input. The Hill-based muscle model (HMM) is the model most frequently used to achieve continuous EMG recognition [9–11]. Buchanan et al. [12] proposed a method of forward dynamic neuromusculoskeletal modeling of the human elbow joint based on EMG, which includes muscle activation kinetics, HMM-based muscle contraction dynamics, elbow musculoskeletal geometry, and forward dynamics for the elbow joint. In addition, an effective tuning method for parameter identification was used to identify physiological parameters [12]. Han et al. [7] used an extended Kalman filter to improve the accuracy of the continuous motion estimation of the human elbow joint, which involved extracting sEMG features to establish a measurement equation, enabling the establishment of a state space with a “physiological motion” equation of the elbow joint. The feedback mechanism improved the robustness of the estimation model. Based on the HMM, Shao et al. [13] proposed the use of muscle internal viscous force to estimate human joint moment by examining the physiological structure of muscle. In simulations, although its effect on the results was much weaker than the noise of sEMG signals, Shao et al.’s [13] method more fully illustrated the mechanism of the musculotendinous force. On the basis of Shao’s study, Pau et al. [14] established a skeletal muscle model of the human upper limb and improved the estimation of elbow joint motion. However, the skeletal muscle model did not fully match the skeletal muscle structure of the human body.

On the basis of Pau’s study [14], we focused on the physiological structure of the skeletal muscles, proposing a new geometric model of the human upper limb. This method better reflects the physiological structure and avoids the need for measuring a large number of unknown physiological parameters from human specimens based on anatomy. Moreover, the proposed approach enables simplification of the required calculation and improved estimation accuracy.

**Fig. 1:**
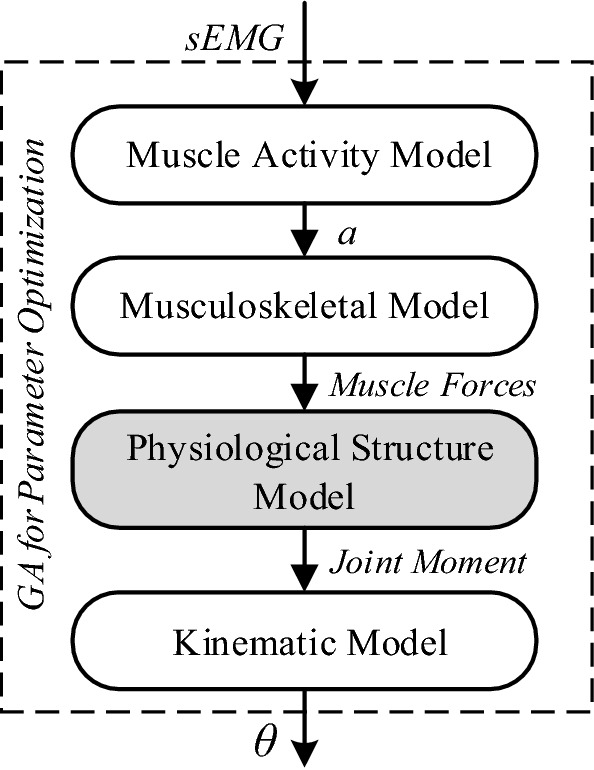
Overview of the continuous motion estimation model (CMEM)

We establish a visualized structural model of the human elbow joint by examining physiological structure. We use this model as a basic framework and establish the relationship between original sEMG signals and joint angles that vary with time. First, a muscle biomechanical model is established with muscle activity as the input. Second, a dynamics model of the elbow joint is established with the muscle biomechanical force as the input, and a genetic algorithm is simultaneously used to optimize the parameters. Finally, through extensive trials, we verified the reliability of joint continuous motion estimation based on the physiological model by results analysis and parameter comparison. An overview of the study is shown in Fig. 1.

## Methods

### Physiological structure

The bony complex of the elbow is an intricate mechanical system comprising the forearm bones (radius and ulna) and the upper arm bone (humerus).

From the perspective of anatomical medicine, the muscles associated with elbow flexion/extension include the biceps, triceps, brachioradialis, brachialis, and anconeus. During movement, the brachioradialis and anconeus play coordinating roles, whereas the biceps and triceps are the main muscles responsible for elbow joint flexion and extension, respectively [14, 15], which is one of the reasons we examined the biceps and triceps in the current study. In addition, the brachialis, which is a deep muscle, was initially taken into consideration. However, according to the previous study [16], during elbow joint extension, the brachialis is only a small muscle antagonist to the triceps, and the sEMG signals produced by the brachialis are difficult to measure. Thus, we did not include the brachialis in the analysis. Taking these factors into consideration, we focused on the biceps and triceps as the main research targets in the current study.

**Fig. 2:**
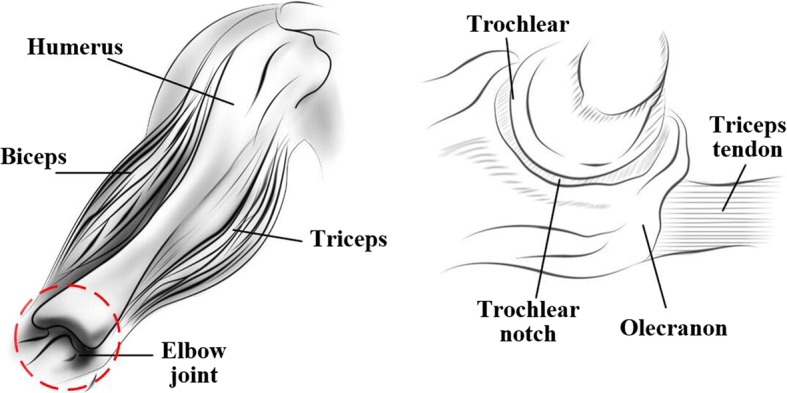
The distribution of muscle groups associated with the upper limb and a lateral drawing of the elbow joint

To establish a physiological model of the elbow, it is necessary to understand the distribution of the major muscle groups: the long head of the biceps originates from the supraglenoid tubercle while the coracoradialis originates from the coracoid process of the scapula. Both muscles converge on the muscle belly and integrate to become the biceps tendon that inserts on the radial tuberosity. The triceps has three heads; the long head originates on the scapula and the two short heads originate from the humerus. They integrate to become the triceps tendon that inserts on the olecranon, as shown in Fig. 2.

The elbow joint comprises the trochlea on the humeral side and the trochlear notch and olecranon process on the ulnar side. The trochlea is a hyperboloid-shaped cylinder that fits well into the trochlear notch [17]. Because of these structures, the elbow joint can be modeled as a hinge joint [14, 15, 18].

**Fig. 3:**
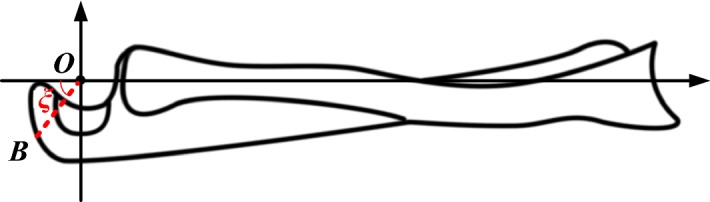
Physiological structure of the forearm

The physiological structure of the forearm comprises the ulna and radius. The tendinous insertion of the triceps averages 20–24 mm by 8–12 mm and extends to the medial margin of the olecranon [19] (see the right side of Fig. 2). We labeled the insertion of the triceps as point *B*. The angle between the straight lines connected by the elbow joint’s center of rotation (*O*), point B, and the axis of the forearm is denoted as *ξ* (see Fig. 3).

**Fig. 4:**
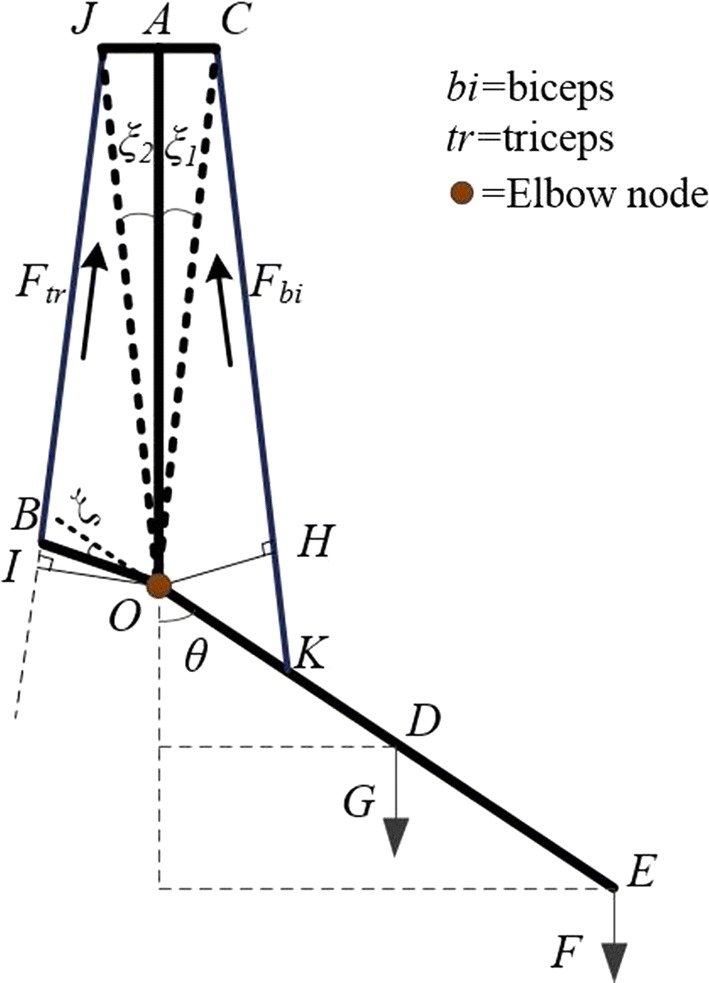
Physiological structural model of the elbow joint

The physiological characteristics described above are important for understanding elbow movement and form the basis of our mathematical model. We established a physiological structural model of the elbow joint to solve the dynamics model (see Fig. 4). This model comprises an equivalent line of muscle fibers and bones.

In Fig. 4, *J* and *C* represent the equivalent origination points of the biceps and triceps on the shoulder, respectively; *AO* and *BOE* represent the humerus and the bones of the forearm, respectively; and *F*_*bi*_ and *F*_*tr*_ represent the force of the biceps and triceps, respectively.

To simplify the complexity of the structural model, we assumed that the axis of the humerus and forearm create an angle of 0° when the elbow is fully extended [20, 21]. Thus, *ξ* = *ξ*_2_. *l*_*OH*_ and *l*_*OI*_ are the arms of the biceps and triceps muscle forces, respectively, which can be obtained using a geometric model.

### Musculotendinous force

**Fig. 5:**
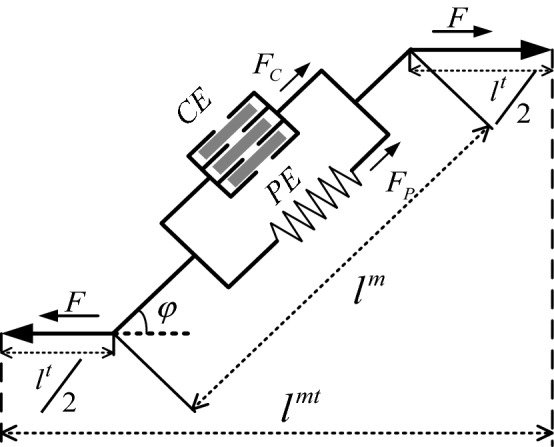
Hill-based muscle model (HMM)

On the basis of the HMM, each musculotendon unit is modeled as a muscular unit with two parallel elements: an active contractile element (CE) producing the active muscle force *F*_*c*_ and a nonlinear passive elastic element (PE) producing the passive force *F*_*p*_, as shown in Fig. 5 [10].

From the HMM, we realize that the muscle force *F* is produced by the combined effects of the muscle contractile force and muscle passive force [12], as expressed by:

1 $$F = (F_{C} + F_{P} )\cos \varphi$$

where *F*_*c*_ is the muscle contractile force, *F*_*p*_ is the muscle passive force, and $\varphi$ is the pennation angle. The optimal pennation angles of the biceps and triceps are no more than 10° [22]. When the optimal pennation angle is 10°, the relative change in the rate of the muscle force is approximately 0.047, which shows that the pennation angle has little effect on the muscle force. This can be written as $\varphi = 0$, and, thus, $\cos \varphi = 1$ [14, 23].

The model of the muscle contractile force and muscle passive force is expressed as [10–12]:

2 $$\left\{ \begin{array}{l} F_{c} = f_{A} (l) \cdot f_{V} (v) \cdot a(k) \cdot F_{{\rm max} } \\ F_{p} = f_{P} (l) \cdot F_{{\rm max}} \end{array} \right.$$

where *f*_*A*_(*l*), *f*_*V*_(*v*), and *f*_*p*_(*l*) are, respectively, the normalized muscle contractile force–length relationship, the muscle contractile force–velocity relationship, and the passive elastic force–length relationship. We take *f*_*V*_(*v*) = 1 [7, 24, 25], and *a*(*k*) is the muscle activation at time *k*. *F*_*max*_ is the maximum isometric muscle force while *l* is the normalized muscle fiber length, which is equal to the current muscle fiber length *l*^*m*^ divided by the optimal muscle fiber length *l*_*0*_^*m*^:

3 $$l = \frac{{l^{m} }}{{l_{0}^{m} }}$$

According to the HMM, the length of the skeletal muscle unit can be calculated as:

4 $$l^{mt} = l^{t} + l^{m} \cdot \cos \phi$$

From this equation, we can calculate the current length of muscle fiber *l*^*m*^. *l*^*t*^ is the length of the tendon, which can be regarded as a constant. *l*^*mt*^ is the length of skeletal muscle, which can be calculated using a geometric model of the human upper limb (see Fig. 4). The skeletal muscle lengths of the biceps and triceps are calculated as:

5 $$\left\{ \begin{aligned} l_{b}^{mt} &= \sqrt {l_{OK}^{2} + l_{OC}^{2} - 2l_{OK} \cdot l_{OC} \cdot \cos (\pi - \theta - \xi_{1} )} \hfill \\ l_{t}^{mt} &= \sqrt {l_{OB}^{2} + l_{OJ}^{2} - 2l_{OB} \cdot l_{OJ} \cdot \cos \left( {\theta - \xi_{2} + \xi } \right)} \hfill \\ \end{aligned} \right.$$

Thus, we established the musculotendinous model. Therefore, when determining the constants *F*_*max*_, *l*_*t*_, and *l*_*0*_^*m*^, we can calculate *l*^*m*^ with (4), *l* with (3), and *F* with (1).

### Prediction model

When the upper limb around the elbow joint is involved with rotation, it can be regarded as a fixed axis rotation of the forearm. The total torque can then be calculated as:

6 $$T = F_{bi} \cdot l_{OH} - F_{tr} \cdot l_{OI} - M_{F} - M_{G}$$

where *M*_*G*_ is the gravitational moment of the forearm and hand acting on the elbow joint while *M*_*F*_ is the external moment acting on the elbow joint.

Assuming that the moment of inertia of the forearm (including the forearm, hand, and load) is *J*, we have the kinetic equation:

7 $$J \cdot \ddot{\theta }_{k} = T_{k}$$

where $\ddot{\theta }_{k}$ is the angular acceleration and *T*_*k*_ is the total torque at time *k*.

We can then obtain the elbow joint dynamics model in discrete time through joint dynamics analysis:

8 $$\left\{ \begin{aligned} &\dot{\theta }_{k + 1} = \dot{\theta }_{k} + \ddot{\theta }_{k} \cdot \Delta t \hfill \\ &\theta_{k + 1} = \theta_{k} + \dot{\theta }_{k} \cdot \Delta t + \frac{1}{2} \cdot \ddot{\theta }_{k} \cdot \Delta t^{2} \hfill \\ \end{aligned} \right.$$

The dynamic model of the elbow joint can be solved using simultaneous Eqs. (6), (7), and (8), which are based on the biceps and triceps muscle force as input and the elbow angle as output.

By combining the three models described above, we obtain the continuous motion estimation model (CMEM) of the elbow joint, which has sEMG signals as input and the elbow rotation angle as output.

## Experiment

### Experimental setup

**Fig. 6:**
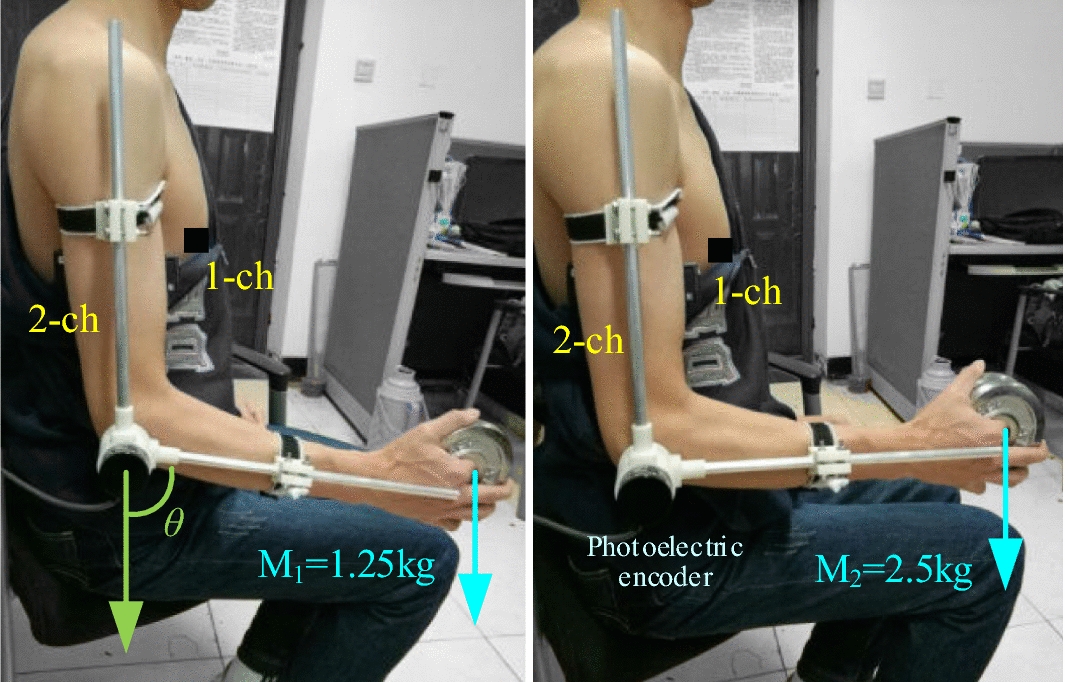
Experimental setup

An experimental method was devised to verify the performance of the CMEM, as shown in Fig. 6. To make the experiment more robust, we selected five subjects with an average age of 25 years to hold loads of 1.25 and 2.5 kg while we examined continuous joint motion. The experiment involved single cycle trials (SCTs), typical cycle trials (TCTs), gradually increasing amplitude trials (GIATs), and random movement trials (RMTs). We performed five sets of tests for each type of trial in each subject. Subjects rested for 5 min between the sets of tests to avoid fatigue.

Before the application of sEMG electrodes, the skin surface was cleaned by rubbing it with alcohol to remove dirt. A Delsys wireless acquisition module (Natick, Massachusetts, USA) was used to collect sEMG signals at a sampling frequency of 2000 Hz. The elbow joint angle was measured with an incremental photoelectric encoder sensor, using the STM32F4 development board for angle capture, and the capture frequency was set to 400 Hz. In the course of the experiment, each subject was asked to keep their arm as relaxed as possible and their upper arm in a vertical position. The subject maintained a smooth and continuous rotating speed of the elbow joint.

### Data processing

The acquired raw sEMG signals were preprocessed to obtain the preprocessed sEMG signals. First, the raw sEMG signals were filtered using a second-order Butterworth filter with a cutoff frequency of 20 Hz for high-pass filtering. Full-wave rectification was then performed. A fourth-order Butterworth filter with a cutoff frequency of 4 Hz was then used for low-pass filtering. Normalized signals were finally obtained as the results divided by the maximum voluntary contraction (MVC) of the muscle.

The MVC is an sEMG measurement corresponding to maximal contraction without causing pain or discomfort [14]. The testing process of MVC has been reported in previous studies [15, 26]. We referred to these methods for testing MVC in the current study. After each subject completed all trials, the MVC for each muscle group was recorded and averaged. Three elbow joint positions (i.e., the angles between the forearm and the ground were approximately 30°, 60° and 90°) were tested. At each position, the subject gradually increased the flexion or extension torque to its maximum and remained at that level for approximately 2 s. The motions of maximum isometric voluntary flexion and maximum isometric voluntary extension were conducted alternately, and verbal encouragement was given for each trial. The subjects had an adequate recovery period between contractions to avoid fatigue. We selected the maximum myoelectric amplitude in these three positions. The trials were repeated three times, and we took the average as the final result for each subject.

The sampling frequency of the joint angle was 400 Hz, while the sampling frequency of muscle activity was 2000 Hz, the same as that of the sEMG signals. Therefore, to ensure that the measured angle and muscle activity had the same sample size, we used a 5-ms time window to calculate the mean muscle activity:

9 $$a_{k} = \frac{1}{5}\sum\limits_{j}^{5} {v_{j,k} }$$

where *a*_*k*_ is the average muscle activation of the *kth* time window and *v*_*j,k*_ is the *jth* original muscle activation in the *kth* time window.

### Model parameter identification

In the simulation, there were 17 undetermined parameters of the CMEM, which were difficult to measure directly. The model parameters of the elbow joint vary between individuals because of individual body differences. It is therefore necessary to identify the relevant parameters before the model is applied to a specific object.

In the current study, we used a genetic algorithm to identify model parameters. The purpose of parameter identification was to find a set of optimal parameters by adjusting the parameters of the CMEM, so that the joint angles *θ* obtained by the model are as close as possible to the real values. The genetic algorithm was used to optimize the unknown physiological parameters, which strongly affect the output or closely related to structural models. The optimization objective function was:

10 $$\hbox{min} \sum\limits_{1}^{n} {(\theta_{ci} - \theta_{mi} )^{2} }$$

where *θ*_*ci*_ is the actual measured angle and *θ*_*mi*_ is the angle of model estimation at time *i*.

**Fig. 7:**
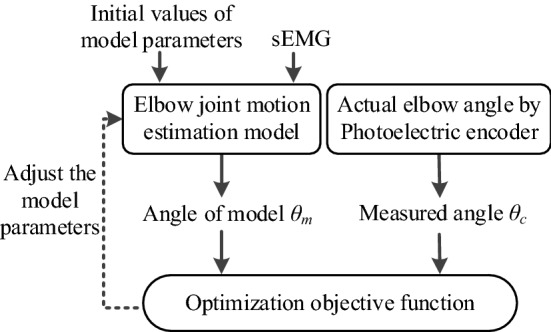
The process of parameter identification based on a genetic algorithm

The initial population evolves in the optimization process, and we used results reported in the literature [23, 27] as a reference. This not only reduced the search space and increased the optimization speed but also eliminated parameter values that deviated significantly from the actual situation (i.e., those that were inconsistent with anatomical knowledge). A decrease in the value of the objective function was taken as the direction of evolution. The optimization process is shown in Fig. 7. Using model parameter identification, we acquired a set of optimal model parameter values that were closest to the actual joint angles. After completing an experiment, we calculated the root-mean-square error (RMSE) of the estimated angles relative to the measured angles to indicate accuracy.

## Results

**Fig. 8:**
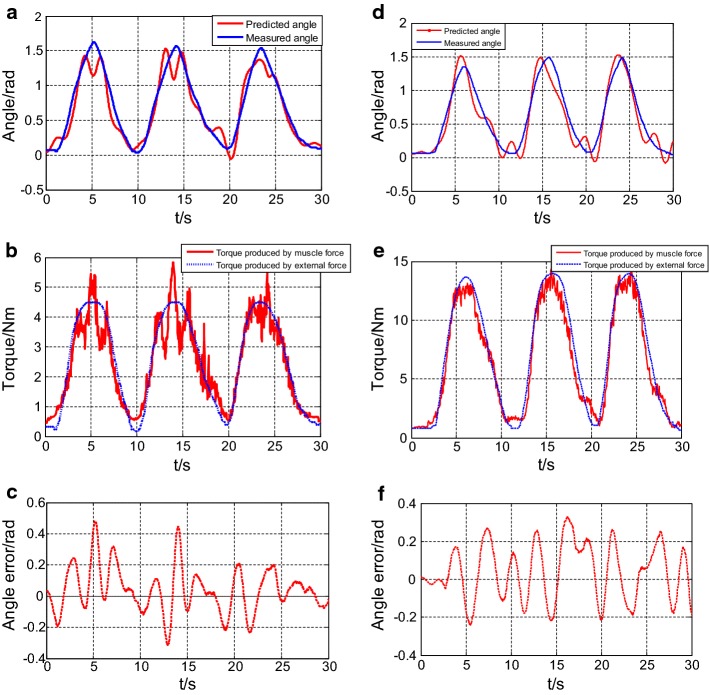
**a** Comparison between the real and estimated motion with RMSE was 0.19 rad (1.25-kg load). **b** Comparison between the real and estimated torque results with 1.25-kg load. **c** Estimated angles error when the load was 1.25 kg; **d** comparison between the real and estimated motion with RMSE was 0.18 rad (2.5-kg load); **e** comparison between the real and estimated torque results with a 2.5-kg load. **f** Estimated angles error when the load was 2.5 kg

Figure 8 shows the relevant results of the TCTs, which are taken as an example of the performance of the prediction model. Panels (a) and (d) show one group of the motion results of TCTs with 1.25 and 2.5 kg loads, with RMSEs of 0.19 and 0.18 rad, respectively. The red line represents the estimated angles, which were the simulation results of the CMEM. The blue line represents the measured angles, which were collected by the photoelectric encoder. The trends of estimated angles and measured angles were largely consistent.

Panels (b) and (e) show the corresponding torque results obtained with 1.25 and 2.5 kg loads, respectively. The red line represents the torque produced by the muscle force and the blue line represents the torque produced by the external force. The difference between them determines the angle acceleration of the elbow joint. In other words, the difference reflects the stability of the predictions. This finding indicates that the experimental results for 2.5 kg were more stable than those for 1.25 kg in the current sample. It can be seen in Fig. 8c, f that the error range for 1.25 kg was greater than that for 2.5 kg, with values of − 0.30 to 0.47 rad and − 0.23 to 0.33 rad, respectively.

**Fig. 9:**
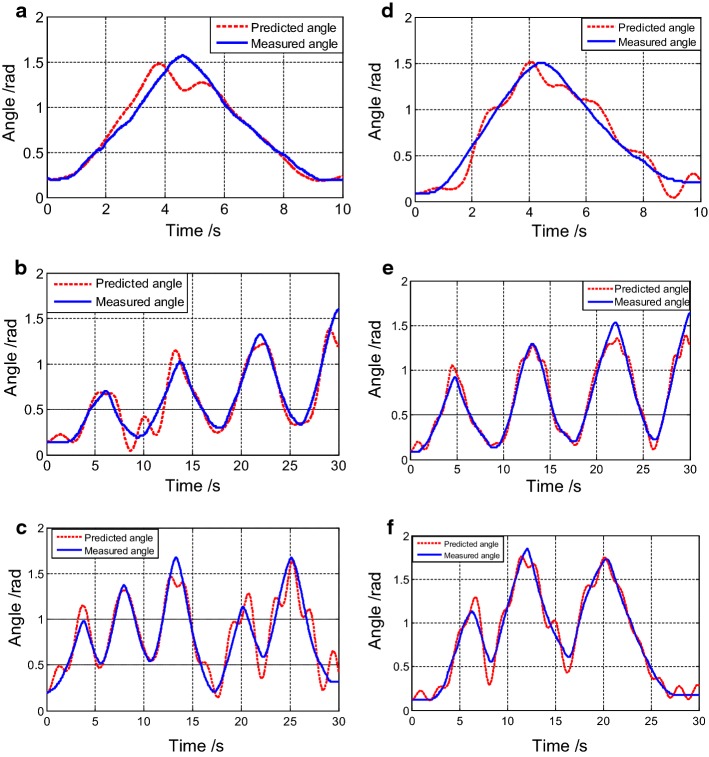
**a**, **d** Are results of the SCTs with 1.25-kg and 2.5-kg loads, RMSEs were 0.12 rad and 0.11 rad, respectively. **b**, **e** Are results of the GIATs with 1.25-kg and 2.5-kg loads, RMSEs were 0.19 rad and 0.17 rad, respectively. **c**, **f** Are results of the RMTs with 1.25-kg and 2.5-kg loads, RMSEs were 0.20 rad and 0.19 rad, respectively

The estimated results of the CMEM after tuning for SCTs, GIATs, and RMTs are shown in Fig. 9. Table 1 summarizes average RMSE values of each set of trial results across all five subjects. The total average is the average RMSE for all subjects. The total averages for 1.25 kg were 0.12, 0.22, 0.22, and 0.26 rad while the averages for 2.5 kg were 0.12, 0.23, 0.24, and 0.25 rad for SCTs, TCTs, GIATs, and RMTs, respectively.

**Table 1:** Summary of average RMSEs and standard deviations of each type of trial results

Sub	SCT		TCT		GIAT		RMT	
A	0.12 ± 0.02	0.10 ± 0.02	0.20 ± 0.03	0.21 ± 0.04	0.19 ± 0.03	0.18 ± 0.03	0.22 ± 0.04	0.19 ± 0.03
B	0.13 ± 0.03	0.12 ± 0.03	0.25 ± 0.04	0.27 ± 0.07	0.26 ± 0.04	0.29 ± 0.06	0.32 ± 0.07	0.33 ± 0.04
C	0.13 ± 0.03	0.11 ± 0.02	0.19 ± 0.03	0.21 ± 0.03	0.21 ± 0.03	0.24 ± 0.04	0.24 ± 0.04	0.23 ± 0.03
D	0.12 ± 0.02	0.13 ± 0.04	0.24 ± 0.06	0.23 ± 0.04	0.25 ± 0.05	0.25 ± 0.04	0.28 ± 0.06	0.27 ± 0.05
E	0.12 ± 0.03	0.11 ± 0.03	0.21 ± 0.03	0.20 ± 0.03	0.19 ± 0.03	0.21 ± 0.03	0.24 ± 0.04	0.24 ± 0.05
Overall	0.12 ± 0.02	0.12 ± 0.03	0.22 ± 0.04	0.23 ± 0.05	0.22 ± 0.05	0.24 ± 0.05	0.26 ± 0.06	0.25 ± 0.06

The unit is rad; *Sub* subject; Each type of trial included the results of 1.25-kg and 2.5 kg-loads

## Discussion

A previous study by Artemiadis et al. [28] reported that RMSEs of the joint angle prediction ranged from 0.03 to 0.16 rad, but the range of arm motion was limited to the horizontal plane. Han et al. [7] used the filtering algorithm of an extended Kalman filter, achieving RMSEs of the typical continuous cycle motion of the elbow joint of 0.11–0.13 rad. Koo et al. [15] established a motion estimation model for the elbow joint and achieved RMSEs of single flexion and single extension movements of 0.61 and 0.33 rad, respectively. Pau et al. [14] established a physiological elbow joint model, reporting RMSEs of 0.11, 0.38, 0.34, and 0.39 rad, respectively, for SCTs, TCTs, GITs, and RMTs. Similar to these previous results, the average RMSEs in the current study ranged from 0.12 to 0.26 rad.

We established a CMEM based on the physiological structures of the elbow joint. The physiological characteristics of the elbow joint were mainly characterized by the geometric model of the upper limb. In the proposed model, the distance between points *J* and *C*, which were the equivalent origination points of the biceps and triceps in the shoulder, should be emphasized. These distances affect the accuracy of the model estimation results, especially the length of *l*_*AC*_. If *l*_*JC*_ is ignored (i.e., *ξ*_*1*_ = *ξ*_*2*_ = 0), *l*_*OH*_ and *l*_*OI*_ will be zero when the joint angle *θ* is zero. This means that the arms of the biceps and triceps force are equal to zero. In such a case, the elbow would be in the dead position, requiring an external force to bend. This does not meet the required characteristics of elbow joint physiological movement. When we considered the *l*_*JC*_, the prediction accuracy and the rationality of the model were significantly improved.

During the course of the experiment, the sEMG amplitude for the biceps was significantly higher than that for the triceps. In the optimization process, the model parameters associated with the biceps were more effective than those associated with the triceps. Table 2 lists the average values of the optimized geometric model parameters obtained by the genetic algorithm for subject *A* with each movement type. It can be seen that *l*_*AC*_ and *l*_*OK*_ had stable ranges of 0.092–0.1 and 0.023–0.04 m, respectively. However, *l*_*AJ*_ and *l*_*OB*_ fluctuated greatly, with *l*_*AJ*_ ranging from 0.021 to 0.096 m and *l*_*OB*_ ranging from 0.013 to 0.092 m. These findings indicate that *l*_*AC*_ and *l*_*OK*_ had a greater effect than *l*_*AJ*_ and *l*_*OB*_ on the accuracy of the experimental results. Using the total average value of *l*_*AC*_ and *l*_*AO*_, we calculated the average value of *ξ*_*1*_ to be 0.44 rad. Thus, *l*_*AC*_ appears to play an important role in elbow movement and should not be ignored.

**Table 2:** The average values of optimized geometric model parameters by GA

Movement types^a^	l_AO_/m	l_AC_/m	l_AJ_/m	l_OE_/m	l_OK_/m	l_OB_/m	l_0_^m^/m	l^t^/m	m/kg	J/kg m^2^
SCT	0.225	0.092	0.092	0.36	0.024	0.092	0.2	0.1	1.94	0.522
	0.231	0.096	0.021	0.44	0.035	0.081	0.2	0.1	1.93	0.811
TCT	0.238	0.1	0.092	0.41	0.028	0.045	0.2	0.1	1.85	0.722
	0.220	0.098	0.067	0.50	0.04	0.069	0.2	0.1	2	0.906
GIAT	0.222	0.096	0.035	0.37	0.023	0.013	0.2	0.1	1.95	0.554
	0.234	0.1	0.059	0.42	0.038	0.086	0.2	0.1	1.97	0.846
RMT	0.223	0.092	0.096	0.41	0.027	0.094	0.2	0.1	2	0.722
	0.224	0.096	0.024	0.35	0.025	0.071	0.2	0.1	2	0.824
Average	0.227	0.096	0.061	0.41	0.03	0.069	0.2	0.1	1.96	0.628
										0.847

This table omits the optimized muscle activity model parameters and each experiment includes the results of 1.25-kg and 2.5-kg loads

^a^*SCT* single-cycle trials, *TCT* typical continuous-cycle trials, *GIAT* gradually increasing amplitude trials, *RMT* random-movement trials

**Fig. 10:**
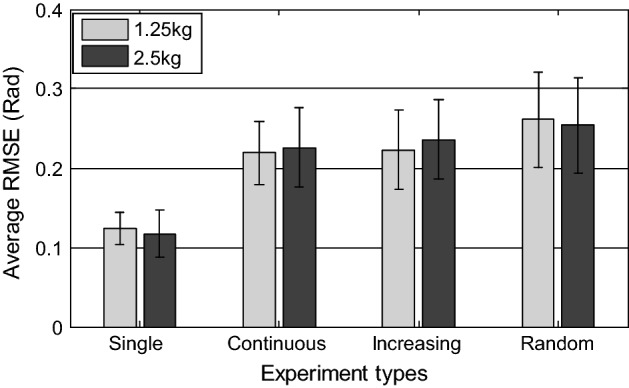
The total average RMSEs for each type of trial

To verify the accuracy and generalizability of the CMEM, extensive trials were required, including comparisons between the four types of trials with 1.25 and 2.5-kg loads. Statistical analysis of the estimated accuracy revealed that the SCTs had smaller average RMSEs (0.12 and 0.12 rad, respectively) and RMSEs became larger as the joint movement became more complex. The total average RMSEs were 0.22 and 0.23 rad, respectively, for TCTs, and 0.22 and 0.24 rad, respectively, for GIATs. The RMTs exhibited the most complex movement and the largest RSMEs (0.26 and 0.25 rad respectively). We plotted a histogram to compare the total average RMSEs of each type of movement when using our proposed method, as shown in Fig. 10. Although subjects were required to rotate the joint as uniformly as possible during the experiments, it was unavoidable that the end acceleration would cause an abrupt change in sEMG signals in the actual movement process and that this acceleration would be more random for complex movement. In addition, although we conducted denoising and feature extraction of sEMG signals in the current study, the sudden change in raw signals still affected the accuracy of the experimental results. Effective feature extraction technology for sEMG signals is therefore essential.

The present study used a genetic algorithm to search for the optimal values of unknown physiological parameters in the CMEM. However, the tuning process inevitably fell into a local minimum. It was thus necessary to increase the number of optimization experiments and identify narrower ranges for parameter searches. This not only reduced the search time but also improved the accuracy of searching for a global minimum. In addition, the genetic algorithm has limitations in terms of practical application, because the optimization process and motion estimation cannot be performed at the same time. Thus, in future studies, we plan to use an online filtering method instead of a genetic algorithm to achieve the real-time prediction of human joint motion. In addition, due to the nature of the sEMG signal, the same movement can be generated by different sEMG signal patterns [14]. The generalization ability of the model optimized by a genetic algorithm therefore has the limitation that model parameters need to be reoptimized for different human bodies and different motion types.

The CMEM of the elbow joint can be applied to the prejudgment of robotic exoskeleton movement, and the motion characteristics of the elbow joint can be applied to bionic joint robots. Currently, the flexibility of the bionic joint is mainly controlled by electric current. It is possible to match the features between sEMG signals and electric current to achieve human-controlled movement of a bionic joint. The establishment of the current model thus has practical significance. A range of barriers must be overcome to achieve such a technology, including the development of methods to reduce of the impact of muscle fatigue in practical applications, avoid the local minimum in the actual optimization by GA, and estimate joint stiffness. These problems must be solved to achieve a user-friendly HRI based on CMEM.

## Conclusion

In the current study, we proposed a visualized structural model of the human elbow joint based on examination of its physiological structure. We used this model as a basic framework and applied an efficient optimization algorithm to establish the relationship between original sEMG signals and joint angle variations along with human movement. This method enabled a better model reflecting the physiological structure and avoided the need to measure numerous human physiological parameters. Compared with previous studies, this approach enabled us to simplify the required calculations and improve the estimation accuracy. The proposed method lays a theoretical foundation for robotic exoskeletons and powered exoskeletons to understand the intentions of human continuous motion.

## References

[1] Hou ZG, Zhao XG, Cheng L, Wang QN, Wang WQ (2016). Recent advances in rehabilitation robots and intelligent assistance systems. Acta Automatica Sin.

[2] Ziai A, Menon C (2011). Comparison of regression models for estimation of isometric wrist joint torques using surface electromyography. J Neuroeng Rehabil.

[3] Nan B, Okamoto M, Tsuji T (2009). A hybrid motion classification approach for EMG-based human–robot interfaces using bayesian and neural networks. IEEE Trans Robot.

[4] Ding QC, Han JD, Zhao XG (2016). Continuous estimation of human multi-joint angles from sEMG using a state-space model. IEEE Trans Neural Syst Rehabil Eng.

[5] Spanias J, Perreault E, Hargrove L (2016). Detection of and compensation for EMG disturbances for powered lower limb prosthesis control. IEEE Trans Neural Syst Rehabil Eng.

[6] Artemiadis P, Kyriakopoulos K (2010). EMG-based control of a robot arm using low-dimensional embeddings. IEEE Trans Robot.

[7] Han JD, Ding QC, Xiong AB, Zhao XG (2015). A state-space EMG model for the estimation of continuous joint movements. IEEE Trans Ind Electron.

[8] Hahne JM, Biebmann F, Jiang N (2014). Linear and nonlinear regression techniques for simultaneous and proportional myoelectric control. IEEE Trans Neural Syst Rehabil Eng.

[9] Cavallaro EE, Rosen J, Perry JC, Burns S (2006). Real-time myoprocessors for a neural controlled powered exoskeleton arm. IEEE Trans Biomed Eng.

[10] Fleischer C, Hommel G (2008). A human–exoskeleton interface utilizing electromyography. IEEE Trans Robot.

[11] Sartori M, Reggiani M, Farina D, Lloyd DG (2012). EMG-driven forward dynamic estimation of muscle force and joint moment about multiple degrees of freedom in the human lower extremity. PLoS ONE.

[12] Buchanan TS, Lloyd DG, Manal K, Besier TF (2004). Neuromusculoskeletal modeling: estimation of muscle forces and joint moments and movements from measurements of neural command. J Appl Biomech.

[13] Shao Q, Bassett DN, Manal K, Buchanan TS (2009). An EMG-driven model to estimate muscle forces and joint moments in stroke patients. Comput Biol Med.

[14] Pau JWL, Xie SSQ, Pullan AJ (2012). Neuromuscular interfacing: establishing an EMG-driven model for the human elbow joint. IEEE Trans Biomed Eng.

[15] Koo TKK, Mak AFT (2005). Feasibility of using EMG driven neuromusculoskeletal model for prediction of dynamic movement of the elbow. J Electromyogr Kinesiol.

[16] Bai J. An elbow biomechanical model based on sEMG. M.S. Thesis, Harbin Institute of Technology, China, 2014.

[17] Rehbinder H, Martin C (2001). A control theoretic model of the forearm. J Biomech.

[18] Rooker JC, Smith JRA, Amirfeyz R (2016). Anatomy, surgical approaches and biomechanics of the elbow. Orthop Trauma.

[19] Jafarnia K, Gabel GT, Morrey BF (2001). Triceps tendinitis. Oper Techn Sport Med.

[20] Kapandji IA (1970). The physiology of the joints.

[21] Amis AA, Dowson D, Unsworth A, Miller JH, Wright V (1977). An examination of the elbow articulation with particular reference to variation of the carrying angle. Eng Med.

[22] Zajac FE (1989). Muscle and tendon: properties, models, scaling, and application to biomechanics and motor control. Crit Rev Biomed Eng.

[23] Pau JWL, Saini H, Xie SSQ, Pullan AJ, Mallinson G. An EMG-driven neuromuscular interface for human elbow joint. In: IEEE Int Conf BioRob. Tokyo, Japan; September 2010. p. 26–9.

[24] Ding QC, Xiong AB, Zhao XG, Han JD. A novel EMG-driven state space model for the estimation of continuous joint movements. In: IEEE SMC Conf. Anchorage, USA; October 2011. p. 2891–7.

[25] Lloyd DG, Bessier TE (2003). An EMG-driven musculoskeletal model to estimate muscle forces and keen joint moments in vivo. J Biomech.

[26] Koo TKK, Mak AFT, Hung LK (2002). In vivo determination of subject-specific musculotendon parameters: applications to the prime elbow flexors in normal and hemiparetic subjects. Clin Biomech.

[27] Holzbaur KRS, Murray WM, Delp SL (2005). A model of the upper extremity for simulating musculoskeletal surgery and analyzing neuromuscular control. Ann Biomed Eng.

[28] Artemiadis PK Kyriakopoulos KJ. EMG-based teleoperation of a robot arm using low-dimensional representation. In: IEEE/RSJ Int Conf Intell Rob Syst. San Diego, CA, USA; 29 Oct.–2 Nov. 2007. p. 489–95.

